# Sulfavant A as the first synthetic TREM2 ligand discloses a homeostatic response of dendritic cells after receptor engagement

**DOI:** 10.1007/s00018-022-04297-z

**Published:** 2022-06-20

**Authors:** Carmela Gallo, Emiliano Manzo, Giusi Barra, Laura Fioretto, Marcello Ziaco, Genoveffa Nuzzo, Giuliana d’Ippolito, Francesca Ferrera, Paola Contini, Daniela Castiglia, Claudia Angelini, Raffaele De Palma, Angelo Fontana

**Affiliations:** 1 Consiglio Nazionale delle Ricerche, Istituto di Chimica Biomolecolare, Via Campi Flegrei 34, 80078 Pozzuoli, Napoli, Italy; 2 Bio-Organic Chemistry Unit, Institute of Bio-Molecular Chemistry, National Research Council, Via Campi Flegrei 34, 80078 Pozzuoli, Italy; 3 Consorzio Italbiotec, Via Fantoli, 16/15, 20138 Milan, Italy; 4 BioSearch Srl., Villa Comunale c/o Stazione Zoologica “A. Dohrn”, 80121 Naples, Italy; 5 Department of Internal Medicine, University of Genova, Viale Benedetto XV 6, 16100 Genoa, Italy; 6 Institute for Applied Mathematics “Mauro Picone”, National Research Council, Via Pietro Castellino 111, 80131 Naples, Italy; 7 Department of Biology, University of Napoli “Federico II”, Via Cupa Nuova Cinthia 21, 80126 Naples, Italy

**Keywords:** Innate immunity, Small molecule, Cellular signalling, Vaccine adjuvant, Neurodegenerative disease, Inflammation

## Abstract

**Objective:**

The immune response arises from a fine balance of mechanisms that provide for surveillance, tolerance, and elimination of dangers. Sulfavant A (SULF A) is a sulfolipid with a promising adjuvant activity. Here we studied the mechanism of action of SULF A and addressed the identification of its molecular target in human dendritic cells (hDCs).

**Methods:**

Adjuvant effect and immunological response to SULF A were assessed on DCs derived from human donors. In addition to testing various reporter cells, target identification and downstream signalling was supported by a reverse pharmacology approach based on antibody blocking and gene silencing, crosstalk with TLR pathways, use of human allogeneic mixed lymphocyte reaction.

**Results:**

SULF A binds to the Triggering Receptor Expressed on Myeloid cells-2 (TREM2) and initiates an unconventional maturation of hDCs leading to enhanced migration activity and up-regulation of MHC and co-stimulatory molecules without release of conventional cytokines. This response involves the SYK-NFAT axis and is compromised by blockade or gene silencing of TREM2. Activation by SULF A preserved the DC functions to excite the allogeneic T cell response, and increased interleukin-10 release after lipopolysaccharide stimulation.

**Conclusion:**

SULF A is the first synthetic small molecule that binds to TREM2. The receptor engagement drives differentiation of an unprecedented DC phenotype (homeDCs) that contributes to immune homeostasis without compromising lymphocyte activation and immunogenic response. This mechanism fully supports the adjuvant and immunoregulatory activity of SULF A. We also propose that the biological properties of SULF A can be of interest in various physiopathological mechanisms and therapies involving TREM2.

**Supplementary Information:**

The online version contains supplementary material available at 10.1007/s00018-022-04297-z.

## Introduction

Pivotal issues in immunology are the ability of the immune system to determine efficient and appropriate responses to pathogens, perform immune surveillance against cancer and clearance of damaged cells. The success of these processes is also dependent on the tolerance to self and the ability to regulate the responses and restore homeostasis once the damage is resolved. Dendritic cells (DCs) are critical effectors of immune system by acting as professional antigen-presenting cells (APCs) [1–3]. Upon activation by engagement of pattern recognition receptors (PRRs), mature DCs stop antigen uptake, start secreting cytokines and increase the expression of surface MHC–peptide complex and co-stimulatory molecules [4]. In this way, DCs integrate signals from the innate and adaptive immune system and shape T cell responses toward either immunity or tolerance [5, 6].

**Fig. 1:**
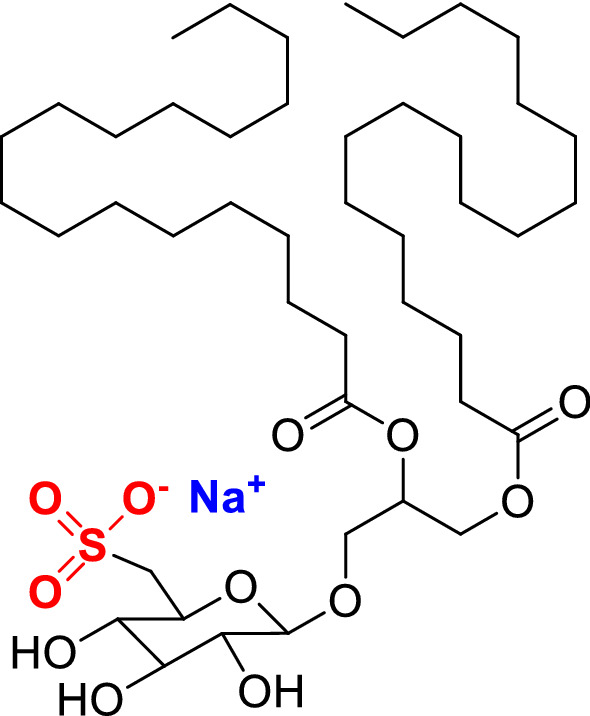
Structure of Sulfavant A (SULF A) with highlighting of the strong anionic sulfonate group and its counter ion

In the last years we reported a novel immunomodulatory sulfolipid named Sulfavant A (SULF A) (Fig. 1), which primes maturation of DCs derived by human monocyte-derived dendritic cells (*h*-MoDCs) through a Toll like receptor (TLR)–independent mechanism [7, 8]. In vivo, the sulfolipid elicited antigen specific production of antibodies and proved efficacy as vaccine adjuvant in an experimental model of melanoma [7]. Khameneh et al. have recently underlined the crucial role of Spleen tyrosine kinase (SYK) and Nuclear Factor of Activated T cells (NFAT) in the DC response to non-TLR–based adjuvants, such as MF59, AS03, and Montanide ISA 720 [9]. The intracellular signaling via SYK has become a paradigm in adapative immunity but more recent studies underline its role outside of this context including several functions of innate immune cells and non-immune functions such as osteoclastogenesis [10]. In these processes, SYK mediates signaling by specific classes of receptors, such as C-type lectins and triggering receptor expressed on myeloid cells-2 (TREM2) [11], widely present on the DC membrane.

Here we aimed to investigate the effect of SULF A on DC maturation by a reverse pharmacology approach starting from the identification of TREM2 as molecular target of the sulfolipid. Although TREM2 has been implicated in a wide array of immune functions especially related to chronic and degenerative diseases, such as Alzheimer disease and dementia [11–14], various aspects of its signalling in other biological contexts remain poorly understood [11]. Thus, this study also offered the opportunity to investigate role and functional aspects of TREM2 engagement in DCs.

## Materials and methods

### Preparation of *T* cells and *h*-MoDCs

Human monocyte-derived dendritic cells (*h*-MoDCs) were obtained from Human peripheral blood from leftover blood bags of healthy volunteers collected in different Blood Transfusion Centers of the Campania Region (Italy). No identifying information on the donors was retained. For each assay, human peripheral blood mononuclear cells (PBMCs) were isolated by routine Ficoll density gradient centrifugation (Ficoll Paque Plus, GE Healthcare, USA). Monocytes were purified from PBMCs using CD14 microbeads (Miltenyi Biotech, Auburn, CA, USA) according to the recommendation of the manufacturer. Purity was checked by staining with a FITC-conjugated anti-CD14 antibody (Miltenyi Biotech, Auburn, CA, USA) and FACS analysis and was routinely found to be greater than 98%. CD14^+^ cells were then cultured for 6 days in RPMI medium supplemented with IL-4 (5 ng/mL) and GM-CSF (100 ng/mL) to differentiate to immature *h*-MoDCs. After harvesting by centrifugation (10 min, 300 g), immature *h*-MoDCs were counted and analysed for CD14^−^/CD83^−^/CD86^+^/HLA-DR^+^ expression by FACS. Purity was routinely > 95%. Immature cells were then seeded in 12 well plates (1∙10^6^ cell/mL) and stimulated with SULF A (10 µg/mL) suspended in PBS at concentration of 1 µg/mL. Stimulation with Pam_2_CSK_4_ 1 µg/mL (Invivogen, San Diego, CA, USA) was used as positive control. Naïve CD4^+^ and CD8^+^ T lymphocytes were isolated from PBMCs by Naïve Pan T Cell Isolation kit (Miltenyi Biotech, Auburn, CA, USA) according to manufacturer instruction. Purity of naïve T cells was confirmed to be > 95% CD45RA^+^/CD45RO^−^/CCR7^+^ by flow cytometry.

### TREM2 reporter assay

2B4 GFP-NFAT reporter T cells stably transfected with human TREM2 cDNAs were a gift of prof. Marco Colonna from Washington University in St. Louis. For the binding assay cells (2 × 10^5^ cells/well) were plated in flat 96-well plate in 0.2 mL/well of RPMI medium and incubated overnight. MeOH was used as solvent to dissolve compounds and by performing the coating of the plate with 0.05 mL of solution of lipids at indicated concentration. Reporter cells activity was assessed after overnight incubation as percentage of GFP^+^ cells subtracted from background (vehicle controls) measured by flow cytometry. Data were acquired by BD C6 ACCURI instruments (BD Bioscience, Frankin Lake, NJ, USA).

### Dectin 1 reporter assay

HEK-Blue™ hDectin-1b Cells (SEAP reporter cells) (Invivogen, San Diego, CA, USA) were plated in 96-well plates at 4 × 10^5^ cells/well in 0.2 mL/well of HEK-Blue™ medium (Invivogen, San Diego, CA, USA) and incubated overnight. MeOH was used as solvent to dissolve compounds and by performing the coating of the plate with 0.05 mL of solution of lipids at indicated concentration. Reporter cells activity was assessed after overnight incubation as quantification of secreted alkaline phosphatase (SEAP) released in the medium by reading the optical density (OD) at 655 nm. Zymosan was used as positive control under the same conditions at concentration of 100 µg/mL according to manufacturer’s instructions. Quantitative data (ng/mL) were obtained by a standard curve for the SEAP protein.

### Flow cytometry

*h*- MoDCs were stained to check the maturation state and the purity with conjugated mouse anti-human HLA-DR BV786, CD83 BUV737, CD86 BV650, CD14 FITC andCD11c PE monoclonal antibodies (BD Bioscience, Frankin Lake, NJ, USA) according to standard protocol. Before acquisition, all cells were incubated with Propidium iodide solution (Thermo Fisher Scientific, Waltham, MA, USA) for dead cell labelling for 5 min at room temperature. Data were acquired by an ARIA or BD C6 ACCURI flow cytometers (BD Bioscience, Frankin Lake, NJ, USA) and analysed with FlowJo 9 software (Tree Star, Inc., Ashland, OR, USA) and BD C6 ACCURI software.

### Real-time PCR

Total RNA was isolated using Trizol Reagent, according to the manufacturer’s protocol. RNA quantity and purity were measured with a NanoDrop 2000 spectrophotometer (Thermo Fisher Scientific, Waltham, MA, USA). Sample purity was checked by A260/A280 ratios between 1.80 and 2.00. Extracted RNAs from all preparations were in this range. Cytokines mRNA expression was measured by quantitative Real-time PCR by using the following primers for DAP12: 5’-GACCCGGAAACAGCGTATCA-3’ (sense), and 5’-GTAGACATCCGACCTCTGACC-3’ (antisense); NFAT1: 5’-ATCTGCAGCATCCCAGTGAC-3’ (sense) and 5’- CGCAGCTCGTAAGAGCCTG-3’ (antisense); NFkβ-p5: 5’- GATCTGCCGAGTGAACCGAA-3’ (sense) and 5’-CAATGTCCTCTTTCTGCACCTT-3’ (antisense); SYK: 5’-CTGGCAGCTAGTCGAGCATT-3’ (sense) and 5’-ACATTTCCCTGTGTGCCGAT-3’ (antisense); RELA: 5’-GAGACACACCACTGCACCTA-3’(sense) and 5’-GCTCAATGACACTGGTCTGC-3’(antisense); TREM2: 5’-GCACTCTCACCATTACGCTG-3’ (sense) and 5’-CTGGTAGAGACCCGCATCAT-3’ (antisense); IL12p40: 5’-GCCCATTGAGGTCATGGTG-3’ (sense) and 5’-TCTTGGGTGGGTCAGGTTTG-3’ (antisense); IL-4: 5’-CGACTGCACAGCAGTTCCAC-3’ (sense) and 5’-CACAGGACAGGAATTCAAGCC-3’ (antisense); 18S: 5’-GGCGACGACCCATTCGAAC-3’ (sense) and 5’-AGGCACGGCGACTACCATC-3’ (antisense); IL-6: 5’-CCCTGAGAAAGGAGACATGTAAC-3’ (sense) and 5’-CCCTGAGAAAGGAGACATGTAAC-3’ (antisense); IL-10: 5’-GGGAGAACCTGAAGACCCTC-3’ (sense) and 5’-AAGAAATCGATGACAGCGCC-3’ (antisense); IFNγ: 5’-ATGGCTGAACTGTCGCCAG-3’ (sense) and 5’-TGCAGGCAGGACAACCATT-3’ (antisense); NFAT2: 5’-GGTTCAGCTCCATGGCTACA-3’ (sense) and 5’-AAGATCTGAAGTCCCAGAGGC-3’ (antisense). 18S Ribosomal RNA (rRNA) was used as a housekeeping gene to normalize sample-to-sample, systematic variation in qPCR assays.

### ELISA

ELISA assays for measuring IL-2, IL-12p70, IFNG, IL-10, IL-4 and IL-23 in supernatants were performed using commercially available kits (Thermo Fisher Scientific, Waltham, MA, USA) following the manufacturer instructions.

### Migration assay of *h*-MoDCs

To assess the migratory capacities of *h*-MoDCs, we used 24-well Transwell microplates (Corning, Amboise, France) with a 8 μm polycarbonate membrane pore size that forbade the passive diffusion, but allowed the active migration, of DCs. For each condition tested, lower chambers of the Transwell (Corning) were filled with 0.6 mL RPMI medium plus nothing (ctrl) and cells (1 × 10^5^) were plated in the upper transwell chamber in 0.1 mL of medium [15]. For the stimulation SULF A was added at concentration of 10 µg/mL, 5 ng/mL LPS and 20 µM NFAT Inhibitor VIVIT (MAGPHPVIVITGPHEE) (N7032, Sigma Aldrich). Cells were allowed to migrate for 3 h at 37 °C. Migrating DCs were harvested from the lower chamber and were counted for 60 s using FACS [16]. Enumerations were performed twice to assess the reliability of the method. We never found more than 10% variation between two counts. Migration index (%) was calculated as the ratio of cells counted from the lower chamber over the total number of cells.

Non migrating cells were removed with cotton swabs, while cells migrated into the lower surface of the filters were stained with crystal violet [17]. Pictures of the migrated cells (purple stained) were taken using a microscope with a 10 × objective.

### Surface binding blocking antibody analysis

Goat anti-human TREM2 antibody (AF1828; R and D Systems, Milan, Italy) was selected as blocking antibody (bAb) according to Quant et al. [18]. Immature *h-*MoDCs were obtained as reported below from 3 donors and incubated for the maturation test with 0.1, 0.2, 0.5 and 1 µg/mL of bAb. Cell concentration was adjusted at 1 × 10^6^ cells/mL and the assay was performed for 24 h in 12-well plates. Cells were then stained for flow cytometry analysis as described above and for TREM2 expression by human TREM2 APC-conjugated antibody (R&D Systems, Milan, Italy). To an aliquot of treated cells (2.5 × 10^5^ cells), biotin-conjugated rabbit anti goat IgG (Dako F0056, Denmark, 1:1000) was added prior to staining by streptavidin for 30 min at 2–8 °C.

### TREM2 neutralization by bAb

Immature *h-*MoDC were prepared from three donors. For the assay, 1 × 10^6^ cells in triplicate for each donor were incubated with 1 µg/mL goat anti-human TREM2 antibody as described before. After incubation at 37 °C for 60 min, 10 µg/mL SULF A was added in triplicate to samples. Cells were kept at 37 °C for 24 h and then harvested and stained as described above for analysis by flow cytometry. As negative control, polyclonal normal goat IgG (isotype control) (AB-108-C; R&D Systems, Milan, Italy) was used at the same concentration of bAb. An aliquot of cells was used for RNA extraction and RT-PCR analysis of IL-12p40 in triplicate as described above.

### siRNA validation

A small interfering oligonucleotide (5’-GGAAGAUGAUGGGAGGAAAtt-3’) that targets the sequence of human TREM2 was purchased by Ambion (Thermo Fisher Scientific, Waltham, MA, USA) together with a non-targeting siRNA (5’-UUUCCUCCCAUCAUCUUCCtt-3’) that was used as negative control. Stock solutions of both siRNA at a concentration of 10 μM were obtained in nuclease-free water. Immature *h*MoDC (1 × 10^6^ cells) were transfected with 50, 100 and 200 nM of siRNA by electroporation using the Amaxa Human Dendritic Cell Nucleofector Kit (Lonza, Basel, Switzerland) following the instructions of the manufacturer and the U-02 Amaxa program (Lonza, Basel, Switzerland). The transfected cells were seeded at 1 × 10^6^ cells/well into 12-well plates and cultured for 24 h. At this time cells were collected for RT-PCR, flow cytometry and vitality by propidium iodide (PI). In a second experiment, immature *h-*MoDCs (1 × 10^6^ cells) were transfected with 200 nM siRNA. The cells were then recovered at 6, 12 and 24 h and analyzed for RT- PCR, flow cytometry and stained with conjugated TREM2-APC antibody (R&D Systems) following the manufacturer’s instructions.

### Interference of TREM2 by siRNA

Immature *h-*MoDCs (1 × 10^6^ cells) were incubated with either 200 nM TREM2 siRNA or 200 nM non-targeting siRNA. Cells were seeded at 1 × 10^6^ cells/well into 12-well plates and cultured 12 h prior to be stimulated by 10 µg/mL SULF A. After other 24 h, treated *h-*MoDCs were recovered and analysed by flow cytometry and gene expression.

### DC costimulation by SULF A and LPS

Immature *h*-MoDCs were prepared and checked for purity as described above. Cells were plated in 12 wells (1∙10^6^ cell/mL) and treated by 10 µg/mL SULF A. After 30 min, 5 ng/mL LPS was added and plates were incubated at 37 °C for further 48 h. After this time, cells and supernatants were recovered and analyzed by flow cytometry, gene expression and ELISA.

### Allogeneic mixed lymphocyte reaction (MLR)

Allogeneic *mixed lymphocyte reaction* (MLR) experiments were conducted using naïve CD3^+^ T lymphocytes (responders) and DCs (stimulators) from seven healthy donors different from whom monocytes were collected. The MLR was performed by seeding 1 × 10^4^ stimulators (DCs) and 1 × 10^5^ responders (T cells) at 1:10 ratio in round bottom 96-well plates in RPMI medium completed with 10% human AB serum. Before co-culture, naïve T cells were counted and labelled by carboxyfluorescein succinimidyl (CFSE) ester assay (Thermo Fisher). After two washes in PBS 5% FBS, cells were used for MLR experiments. Unstimulated CFSE labelled lymphocytes were used to set the gate for CFSE control. Three replicates of stimulation with 10 µg/mL SULF A or 1 µg/mL phytohemagglutinin (PHA) were set for each donor. T cells proliferation was analysed by dilution of the CFSE signal using flow cytometry (FACS ARIA; BD Bioscience, Frankin Lake, NJ, USA).

### Statistical analysis

For all markers analyzed by flow cytometry, isotype controls were used by setting gates between 0.5 and 1% positive events. Statistical analysis to compare the mean values for two groups was performed using non-parametric (two-sample) Wilcoxon test or T test. The paired version of the test was used when replicates were matched in the two conditions. For multiple groups, we used a one-way or two-way ANOVA with repeated measures (RM ANOVA) followed by post-hoc analysis with a series of pairwise T tests. We used BH correction or Tukey test for multiple comparisons. We also fitted linear-mixed models (LMM) when technical replicates were considered. A *p* value less than 0.05 was considered statistically significant. Graphics were drawn by GraphPad Prism 8 (GraphPad Software, San Diego California, USA).

## Results

### SULF A induces selective TREM2 signalling on reporter cell lines

**Fig. 2:**
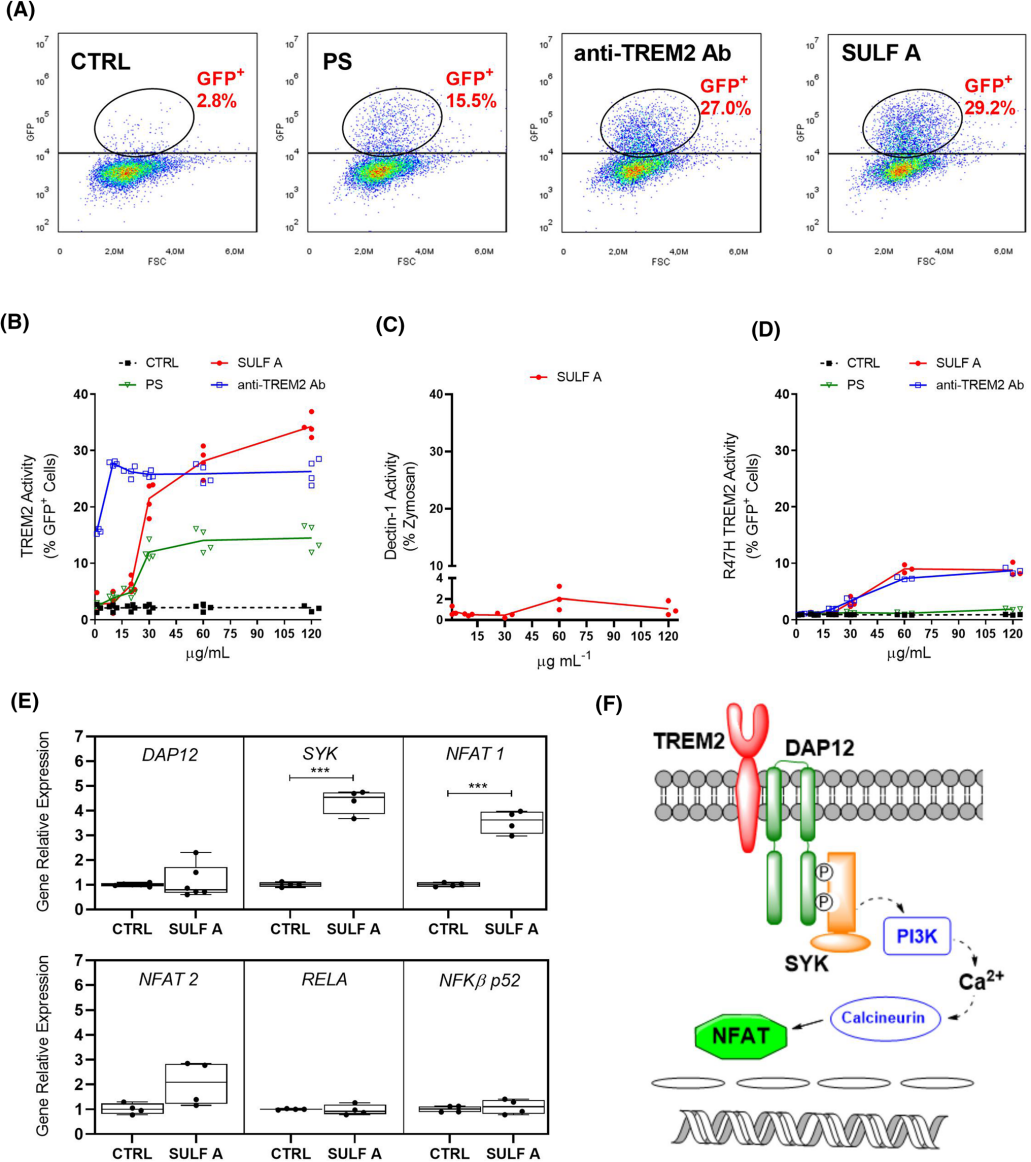
Engagement of SYK-coupled receptors in reporter cells and expression of intracellular TREM2 signaling molecules in *h*-MoDC by Sulfavant. CTRL = untreated reporter cells; SULF A = Sulfavant A; PS = phosphatidylserine; anti-TREM2 Ab = anti human TREM2 antibody. **A** Flow cytometry analysis of GFP expression in human TREM2-reporter cell after stimulation. One representative experiment by 60 µg/mL PS, 60 µg/mL anti-TREM2 Ab, and 60 µg/mL SULF A is shown; **B** Dose-dependent response of human TREM2-reporter cells (*n* = 4). Statistical analysis (see Supplementary Material) was performed using two-way RM ANOVA followed by BH correction in the post-hoc analysis; **C**) Dose-dependent response of Dectin 1-reporter cells (*n* = 3). Statistical analysis was performed using one-way RM ANOVA; **D** Dose-dependent response of mutant R47H human TREM2-reporter cells (*n* = 3). Statistical analysis was carried out as in Fig. 1B; **E** Relative expression of the main genes related to the intracellular TREM2 pathway after 3 h in *h*-MoDC from four donors (*n* = 4). Statistical differences were determined by paired samples T test (one-side alternative). Significance refers to *** *P* < 0.001; **F** Proposed TREM2 signaling pathway with the main steps for the intracellular propagation of the signal via DAP12/SYK upon receptor activation

Since its discovery, the identification of endogenous ligands of TREM2 has proven elusive although there is an emerging pattern of evidence suggesting that the receptor can interact with a wide array of negatively charged molecules mostly associated to tissue damage or cellular stress [19–22]. Except for the reports on the binding of the ubiquitous protein cyclophilin A [23] and a X-ray crystallography model of TREM2 centered on a prominent surface of positively charged residues [24], most studies are related to the affinity of putative ligands with the receptor [11]. For this reason, in order to test the binding of SULF A to TREM2, we used a consolidated TREM2-reporter cell line that transduces receptor engagement by synthesis of GFP [20]. After incubation with SULF A, the receptor activation was measured as GFP^+^ cells by flow cytometry in comparison to a polyclonal anti-TREM2 antibody (Ab) and phosphatidylserine (PS), which is one of the suggested *bona fide* ligands [25] (Fig. 2A). The response was dose-dependent in the range from 1 ng/mL to 120 µg/mL of SULF A with an activity that was higher than that of PS and comparable to anti-TREM2 antibody binding (Fig. 2B). The dose–response curve of the cell-based assay showed a sigmoid shape with an EC_50_ of 35.9 µg/mL (Supplementary Fig. 1). Conversely, in the same range of concentrations, SULF A failed to stimulate reporter cells for CLEC7A (Fig. 2C), a glycoprotein of the C-type lectin family, and R47H TREM2 (Fig. 2D), a variant of TREM2 that is known to weakly bind PS and other lipid ligands in microglia [13, 20, 25]. These results proved that SULF A binds to TREM2 in a selective manner and ruled out the non-specific response of the reporter cells.

### Transcriptional signature of intracellular TREM2 pathway after stimulation by SULF A

The downstream cascade signal of TREM2 is mediated by the electrostatic interaction with DNAX-activation protein 12 (DAP12) [11, 13] that, upon phosphorylation, recruits SYK and initiates the intracellular pathway. The next steps are not fully elucidated but independent authors reported that TREM2-mediated activity depends on NFAT [20, 26, 27], a family of five transcription factors which are mostly regulated by Ca^2+^. In striking contrast to the response to C-type lectins, such as dectins or mincle, that requires NFAT together with canonical and non-canonical pathways of nuclear factor-B (NF-kB) in DCs and macrophages [28], 28], TREM2 signaling transduction is independent of NF-κB in DCs [30]. Thus, in order to provide further indications of the signalling originated by SULF A and to differentiate between the intracellular pathways of TREM2 and Dectins/Mincle, we carried out a transcriptional analysis of the target genes *DAP12*, *SYK*, *NFAT1*, *NFAT2* together with the response of the inducible transcription factors RELA (also called NF-κB p65) and NF-κB p52 that are related to the canonical and non-canonical NF-κB pathways. After 3 h from the stimulation by 10 µg/mL SULF A, *h*-MoDC showed significant upregulation (expression fold change above 3) of *SYK* and *NFAT1*, while transcriptional levels of *NFAT2* were affected to a lesser extent (Fig. 2E). SULF A did not vary the expression levels of TREM 2 (data not shown), which agrees with the unchanged transcription of DAP12 that is the related cytoplasmic signal-transducing element. Furthermore, NF-κB pathway was unaffected by the sulfolipid while *RELA* expression was increased by the addition of the TLR agonists LPS or PAM_2_CSK_4_ (Supplementary Fig. 2). Transcriptional analysis over 24 h revealed the return to basal or near-basal levels of *SYK* and *NFAT1* expression after 6 h from the addition of SULF A (Supplementary Fig. 3). These data provided a support to the downstream pathway depicted in Fig. 2F and, in concomitance with the absence of the NF-κB- dependent release of pro-inflammatory cytokines (see below), underlines a marked difference with DC maturation induced by traditional PRR agonists, like LPS and zymosan.

### Functional response of *h*-MoDCs to SULF A

**Fig. 3:**
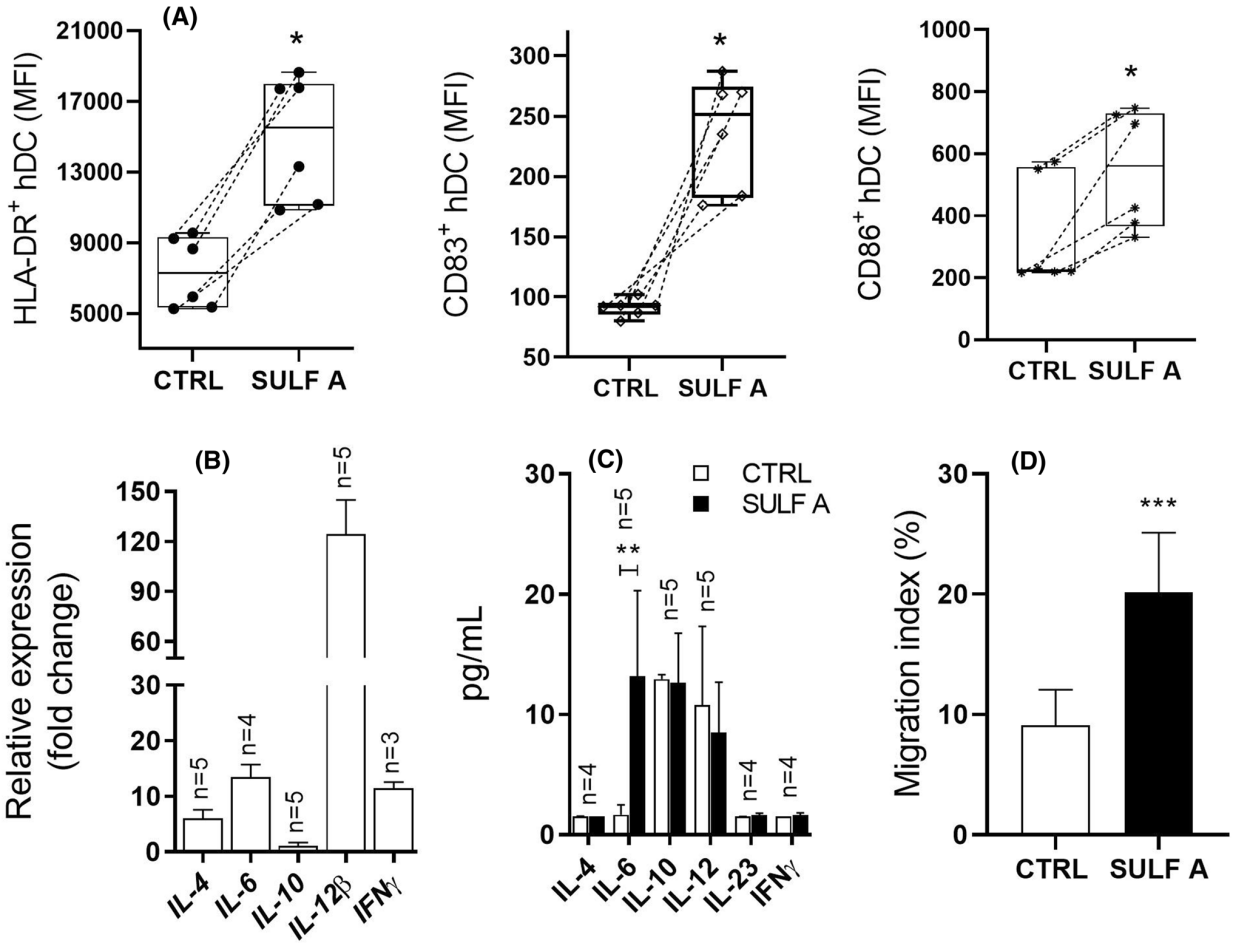
Maturation of *h*-MoDCs following exposure to Sulfavant A. CTRL = untreated cells; SULF A = 10 µg/mL Sulfavant A. **A** Surface expression as Mean Fluorescence Intensity (MFI) of HLA-DR, CD83 and CD86 on six matched donors (*n* = 6) at 24 h by flow cytometry; **B** RT PCR analysis of cytokine gene expression at 24 h; Relative expression is reported as fold changes over ctrl cells; *n* = donor replicates; **C** ELISA analysis of cytokines in the supernatants at 48 h; *n* = donor replicates; **D** In vitro migration assay of *h-MoDCs* untreated (ctrl) and stimulated with 10 µg/mL SULF A for 3 h. Data are expressed as migration index (%) obtained from the ratio between migrated cell counts over the whole cell number. Tests were performed from three donors. Statistical significance (*P < 0.05; **P < 0.01; ***P < 0.001) was established by non-parametric two-sample Wilcoxon Test (two-side alternative). Paired tests and one-side alternatives were used for marker surface expression

We have previously reported that stimulation of *h*-MoDCs by SULF A induces upregulation of HLA-DR and costimulatory molecule CD83, transcription of IL-12p40 (also named IL-12β) and IFN-γ genes after 24 h [7, 8]. *h*-MoDCs are primary cultures derived from human monocytes and widely used as cell model of APC. In line to our previous report, at 24 h stimulation by 10 µg/mL SULF A induced an upregulation of MHC Class II (HLA-DR) and key costimulatory molecules CD86 and CD83 in *h*-MoDCs from 6 human donors (Fig. 3A), with unequivocal evidence on the modulatory effect of the sulfolipid on the costimulus. Within 24 h from the treatment (Fig. 3B), there was the significant increase of the transcription of *IL-12β* and, at lower extent, of *IL-4*, *IL-6*, and *IFNγ* while no signal was detected for *IL-10*, *IL-23p19* and *TGFβ1* (not shown). However, ELISA on the supernatants after 48 h revealed only low and variable secretion of IL-6 [31] without production of Th1-type or Th2-type cytokines (Fig. 3C). This maturation phenotype of DCs is unconventional for the divergence between the marked stimulatory effect on surface molecules related to T cell signalling and the hyporesponsiveness of cytokine patterns related to typical inflammatory or tolerogenic signatures. Thus, we decided to test the effect of SULF A on the migratory activity that is a key function of mature DCs and requires the acquisition of an ameboid-like shape [32]. The migration assay was performed in a cell transwell [33] and showed a significant enhancement of the migration event of the treated cells in comparison to control (Fig. 3D). According to different subsets, DCs do not tend to remain sessile even if their movement is still limited until they undergo maturation [32, 34].

### Neutralization of SULF A activity by antibody blocking of TREM2

**Fig. 4:**
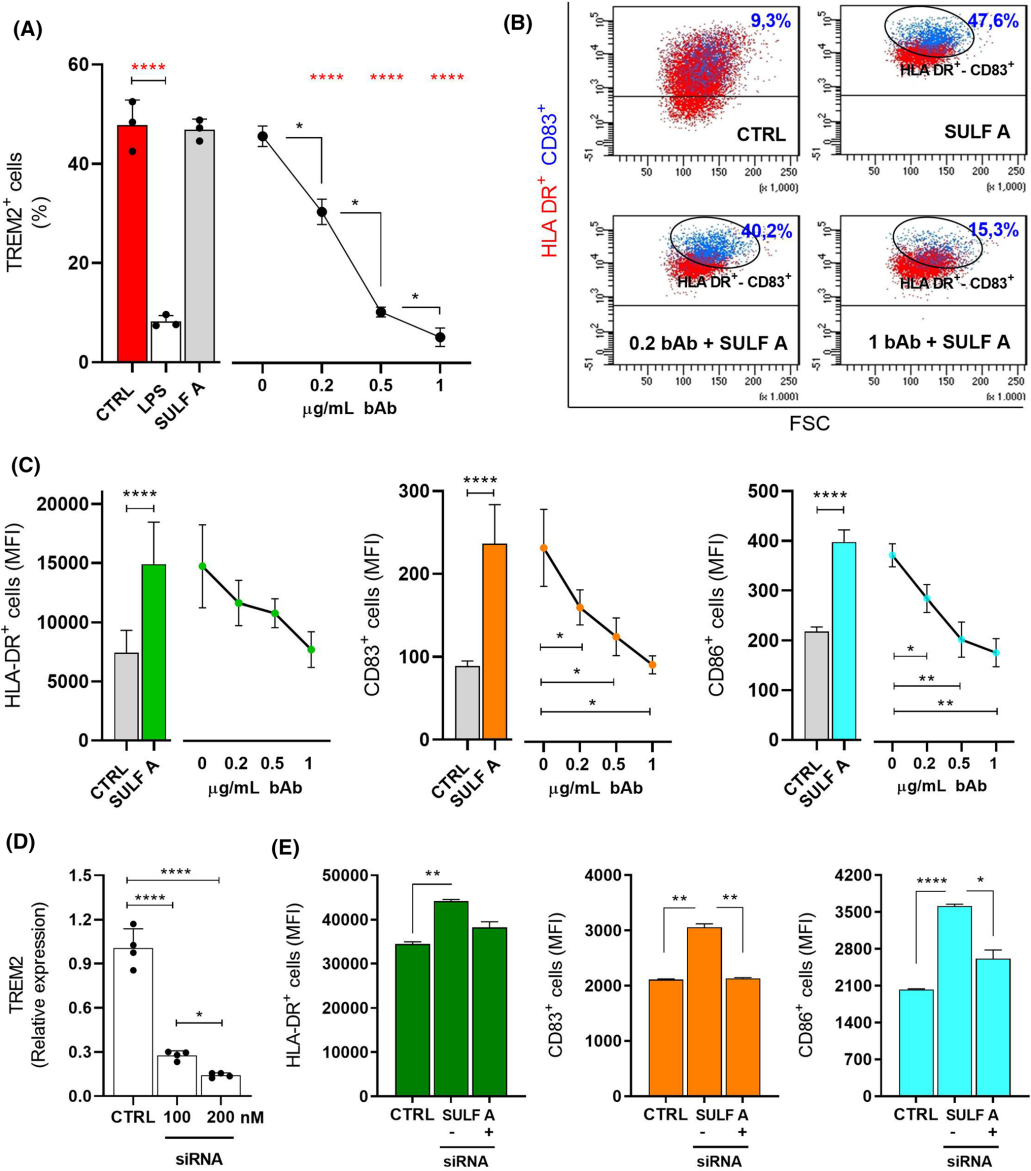
Functional impairment of Sulfavant A activity by antibody blocking assay and gene silencing of TREM2 in *h*-MoDC. CTRL = untreated cells; SULF A = 10 µg/mL Sulfavant A; bAb = anti-TREM2- blocking antibody AF1828. **A** Dose-dependent shedding of TREM2 on h-MoDCs from three donors (*n* = 3) by increasing concentration of the blocking antibody. Effect was compared to 10 ng/mL LPS; **B** Flow cytometry gating analysis of HLA-DR and CD83 expression on *h*-MoDCs treated by sufavant A alone and together with 0.2 µg/mL and 1 µg/mL of anti-TREM2 blocking antibody; **C** Dose-dependent reduction of HLA-DR (right), CD83 (middle) and CD86 (right) on h-MoDCs treated by Sulfavant A and increasing concentration of anti-TREM2 blocking antibody from three donors (*n* = 3); **D** Reduction of TREM2 gene expression by 100 and 200 nM siRNA in *h*-MoDCs from two donors with two technical replicates (*n* = 4); **E** Effect of TREM2 gene silencing on the surface expression of HLA-DR (left), CD83 (middle) and CD86 (right) in h-MoDC by flow cytometry. Cells were silenced by 200 nM siRNA prior to addition of Sulfavant A from four donors (*n* = 4). CTRL = cells treated with 200 nM negative siRNA. Surface expression is reported as Mean Fluorescence Intensity (MFI). Statistical significance (*P < 0.05; **P < 0.01; ***P < 0.001; ****P < 0.0001) was determined by one-way RM ANOVA (panel **A**, **C**–**E**) or LMM (panel **D**) followed by appropriate post-hoc analysis including Dunnett, BH or Tukey's test (see Supplementary Material)

To confirm that TREM2 signalling is responsible for the DC maturation induced by SULF A, we carried out an antibody blocking assay, a widely accepted method to determine the receptors involved in biological response. Cells are preincubated with the blocking antibody (bAb) and then stimulated with the putative ligand, whereby decreased activity against targets is attributed to the blocked receptor. In our case, we used a polyclonal goat anti-human TREM2 IgG (TREM2-bAb) that binds the ectodomain of the protein [18]. Prior to start the experiment with SULF A, the effect of TREM2-bAb was tested on *h-*MoDCs from three donors. The antibody did not induce DC maturation between 0.1 and 1 µg/mL. In this range of concentrations, levels of HLA-DR, CD83 and CD86 were unaffected in comparison to control cells (Supplementary Fig. 4), whereas a dose-dependent reduction of the free receptor on the DC surface was verified by an anti-TREM2 antibody by flow cytometry (Fig. 4A). The occupancy of the receptor on the cell surface of *h-*MoDCs was linear with the increase of the concentration of anti-human TREM2-bAb up to 1 μg/mL. At this concentration, the level of the detectable receptor was as low as the levels observed after treatment of DCs by the TLR-4 agonist LPS, which is known to induce TREM2 suppression in microglia [35]. On the contrary, the immunocytochemical analysis by anti-goat biotin secondary antibody (1:1000) followed by staining with streptavidin confirmed the presence of the TREM2-bAb with an increase of the fluorescence linearly related to the concentration (Supplementary Fig. 5). We next evaluated the inhibitory effects of TREM2-bAb on the expression of MHC II and costimulatory molecules induced by SULF A. As shown in Fig. 4B for HLA-DR^+^CD83^+^ hMoDC cells, flow cytometry analysis revealed that pre-incubation of *h-*MoDCs with the blocking antibody reduced the activity of 10 μg/mL SULF A. The effect was similar on the co-stimulatory molecules CD83 and CD86 (Supplementary Fig. 6) and the statistical analysis on different donors revealed a dose dependent-response with 100% inhibition at 1 µg/mL TREM2-bAb (Fig. 4C). Since the blocking antibody did not initiate TREM2 signalling, the inhibition of SULF A induced DC maturation was due to the non-functional occupation of the receptor. In contrast, treatment with an isotype control did not affect the stimulatory activity of the sulfolipid (Supplementary Fig. 7). These results suggested that the mechanism of action of SULF A is mediated by TREM2 in a specific manner.

### Inhibition of SULF A activity by gene silencing of TREM2

As a further demonstration of the role of TREM2, we tested the correlation between TREM2 expression levels and SULF A-dependent maturation of *h*-MoDCs. First, we verified the efficiency of a TREM2 siRNA to knock down mRNA and protein levels of the receptor. As shown in Fig. 4D, TREM2 expression was significantly reduced in interfered *h*-MoDCs, with a down-regulation that was linearly dependent on the concentration of the interfering oligonucleotide. The effect of transfection was optimal with 200 nM siRNA at 12 h when we observed reduction of TREM2 gene together with no significant change of the cell vitality (over 80%). At this concentration, expression of the phenotypic markers HLA-DR, CD83 and CD86 was unaffected in comparison to both untreated cells and cells transfected with non-targeting siRNA control, thus ruling out non-specific effects (Supplementary Fig. 8). On the other hand, staining by conjugated TREM2-APC antibody showed a reduction of the receptor occurrence on cell surface of *h-*MoDCs interfered with TREM2-siRNA (Supplementary Fig. 9). Incubation of these cells with 10 µg/mL SULF A revealed a clear reduction of the effect of the sulfolipid on the expression of HLA-DR, CD83 and CD86 (Fig. 4E). In analogy with the effect of the blocking antibody reported above, the depletion of the receptor on the cell surface prevented the phenotypic maturation pattern induced by SULF A, which is fully consistent with a TREM2-dependent response of *h-*MoDCs to the sulfolipid.

### Effect of SULF A on TREM2-TLR crosstalk

**Fig. 5:**
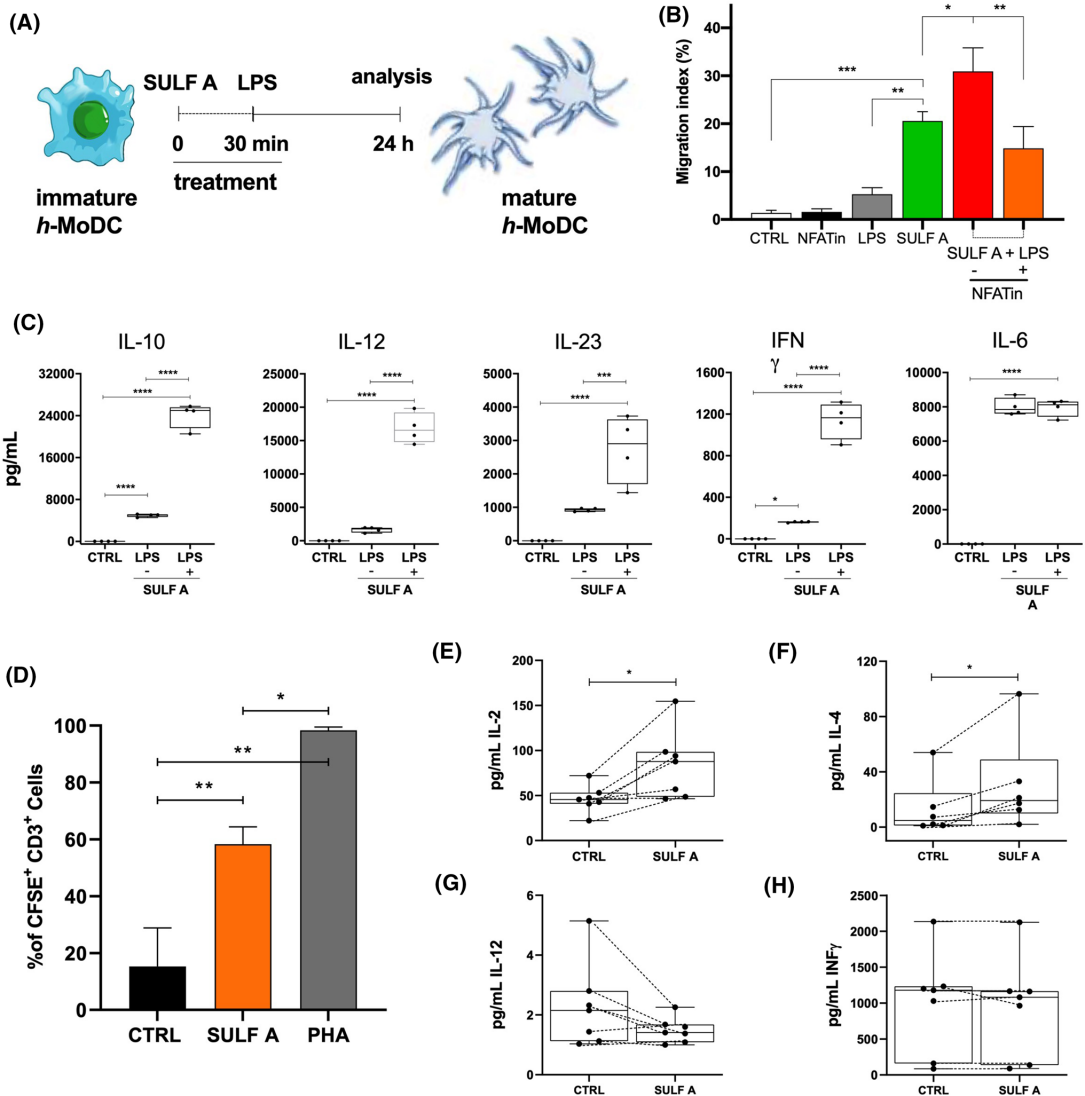
Sulfavant A affects TLR-dependent cytokine release in *h*- MoDCs and allogeneic MLR. CTRL = untreated cells; SULF A = 10 µg/mL Sulfavant A. **A** Design of the co-stimulation experiment by addition of 10 µg/mL SULF A followed by 5 ng/mL LPS; **B** In vitro migration assay of *h-MoDCs* by co-stimulation and in presence or absence of 20 µM NFAT inhibitor (NFATin). Data are expressed as migration index (%) obtained from the ratio between migrated cell counts over the whole cell amount. Tests were performed from four samples. Statistical significance (**P < 0.01; ***P < 0.001; ****P < 0.0001;) was established by non-parametric two-sample Wilcoxon Test (two-side alternative); **C** ELISA analysis of cytokines in the supernatants of the co-cultures after 48 h from two donors with two technical replicates. Statistical analysis was performed by LMM. Post-hoc analysis was carried out using Tukey test; **D** Flow cytometry analysis of T lymphocytes expansion. CFSE-labelled T cells were co-cultured with stimulated *h*-MoDC (1:10 ratio) for 7 days. CTRL = untreated co-colture; SULF A = co-colture stimulated by 10 µg/mL Sulfavant A; PHA = co-colture stimulated by 1 µg/mL phytohemagglutinin (positive control). Proliferation rate is expressed as median values of CFSE^+^ CD3^+^ cells in allogeneic co-cultures from three different donors (*n* = 3). **E**–**H**) ELISA of IL-2, IL-4, IL-12 and IFNγ in the supernatants of the allogeneic co-cultures. Statistical significance (*P < 0.05; **P < 0.01;) was performed in matched samples of different donors (*n* = 7 for IL-2, IL-12 and IFNγ, *n* = 6 for IL-4) by paired samples Wilcoxon Test (two-side alternative). Significance refers to *P < 0.05, **P < 0.01, ***P < 0.001, ****P < 0.0001

TREM2 signalling has acquired relevance in the context of tissue damage and cross-talk with other pathways activated by danger signals [11]. Thus, in order to provide a functional clue on TREM2 activation by SULF A, we tested the molecule for the ability to modify the DC response to TLR agonist. As reported in Fig. 4A, LPS strongly down-regulated TREM2 expression on the surface of *h*-MoDCs. Considering the low amount of residual TREM2, stimulation by SULF A had no detectable effect on these cells (not shown). On the contrary, a 30 min preincubation of *h*-MoDCs with 10 µg/mL SULF A followed by 5 ng/mL LPS (Fig. 5A) downregulated the expression of HLA-DR and the costimulatory markers CD83 and CD86 in comparison to control stimulated only by LPS (Supplementary Fig. 10). Double-treated cells showed a migration activity increased in comparison to both control and treatments with the single stimulus (Fig. 5B). This response was antagonized by the NFAT inhibitor VIVIT (MAGPHPVIVITGPHEE) that selectively and potently interrupts calcineurin docking onto NFAT and the subsequent NFAT dephosphorylation [36], thus providing further evidence of the involvement of this transcription factor in the response of *h*-MoDCs to SULF A. After 24 h, combination of SULF A and LPS also increased production of proinflammatory cytokines IL-12, INFγ, and IL-23 in comparison to LPS alone but also triggered strong synthesis of anti-inflammatory IL-10 (Fig. 5C). On the contrary, sequential addition of SULF A and LPS did not affect the synthesis of IL-6 by *h*-MoDCs (Fig. 5C), as well as suboptimal concentration of the sulfolipid (1 µg/mL) did not modify the response to LPS. IL-10 can directly attenuate inflammatory profile of DC and macrophages by an autocrine mechanism [37, 38], as well as reduce the excessive proinflammatory responses as part of a negative-feedback mechanism to limit an overactive immune response in tissues during infection [39]. Taken together, these data suggest that SULF A tunes DC response to inflammatory signals by a release of IL-10, which is consistent with a mechanism of homeostasis aiming to ensure an appropriate and balanced immune activation.

### T cell priming and differentiation by SULF A-stimulated DCs

Instruction of T lymphocyte response is the hallmark of DC function. Due to the DC ability to modulate activation and function profile of *T* cells, DC represent a main target to improve manipulation of immune responses and vaccine development [40, 41]. In consideration of the unconventional DC phenotype induced by SULF A, we tested the sulfolipid effect on priming and function of T lymphocytes in a mixed lymphocyte reaction (MLR). After excluding direct activation of T lymphocytes by SULF A, *h-*MoDCs (stimulators) and naïve *T* cells (responders) from seven different healthy donors were cocultured to determine T cell proliferation and cytokine release. As shown in Fig. 5D, the addition of SULF A to the MLR increased lymphocyte proliferation in comparison to unstimulated cocultures (control). The effect was comparable to the ex vivo expansion induced by the mitogen phytohemagglutinin (PHA) used as positive control. Measurements of the cytokines in the supernatant of the MLR showed that SULF A upregulated production of IL-2 and IL-4 (Fig. 5E–F). Both molecules are pleiotropic cytokines and key signals in growth, differentiation and in vivo homeostasis of T cells [42, 43], yet their functions can be highly divergent and context dependent. The levels of IL-2 and IL-4 were unaffected by SULF A in T cells alone (Supplementary Fig. 11), thus indicating that their modulation was strictly related to the trigger of SULF A on DC maturation. Furthermore, according to the lack of pro-inflammatory activity by SULF A, we also found that the sulfolipid did not affect release of Th1 cytokines, namely IL-12 and IFNγ, in the MLR system (Fig. 5G–H). These results support the unconventional maturation of DC and the resulting regulatory immune response induced by SULF A through TREM2 engagement.

## Discussion

This study demonstrates that TREM2 mediates the effects of SULF A on DC maturation. In agreement with the regulatory function of this receptor, the stimulation by SULF A prompted the differentiation of DC towards a novel homeostasis-determining phenotype (homeDCs) displaying migratory activity, hypoproduction of cytokines and up-regulation of the costimulatory and class II Ag-presenting molecules (Fig. 3). These features are unique and of interest in the design of DC-based vaccines in immunotherapy since, differently from other maturation methods so far reported for MoDCs, differentiation of homeDCs does not imply acquisition of inflammatory functions.

The response induced by the sulfolipid is correlated to the TREM2 expression on the membrane since both antibody blocking and down-regulation of the receptor transcription inhibited DC maturation in a dose-dependent manner. This loss-of-function is similar to the hyporesponsiveness to putative ligands due to either TREM2 shedding by γ-secretase (also known as sheddase) [44] or to the reduced availability of TREM2 on cell surface by pathogenic mechanisms due to mutations or incorrect glycosylation. Colonna and co-workers first pointed out that TREM2 on microglia can sense negatively charged lipids as an “eat-me” signal for professional phagocytes [20]. In line with this view, two very recent studies report that mycolic acid derivatives and aminophospholipids, such as phosphatidylserine (PS) and phosphatidylethanolamine, can trigger TREM2 signalling in apoptotic cells and macrophages [21, 22]. SULF A (Fig. 1) is a sulfoquinovoside glycerolipid with two saturated fatty acid chains that resemble phospholipids of bacterial membranes [19]. The sulfoquinovoside group mimics the polar head of phospholipids but the strong acidic property of the sulfonic acid increases the negative charge density in comparison to phospholipids. According to the current model of binding to TREM2 [20, 45], this is expected to promote the interaction with the receptor thus explaining the stronger signal compared to PS and other *bona fide* ligands (Fig. 2B). The R47H variant of TREM2 shows a rare mutation within the ligand-binding region of the receptor, which abrogates the response to PS and other putative ligands [20, 24] and increases the risk of neurodegenerative pathologies as Alzheimer disease [12, 46]. The reduced affinity of SULF A for the mutant receptor (Fig. 2D) is clear evidence of the capacity to structurally mimic the binding affinity of endogenous ligands. It is also worth noting that the sugar head of SULF A resembles the structure of the cyclic polyol pinitol, a natural product of pharmacological interest that is suggested to ameliorate inflammation by binding to TREM2 in macrophages and microglia [47].

Several mechanisms have been proposed to explain transcriptional activation downstream of TREM2 engagement but the absence of specific ligands so far hampered the characterization of the receptor signal. SULF A induced a transient up-regulation of the expression of SYK and NFATc genes within 3 h from the stimulation, with a transcriptional signature that was very different from that induced by a TLR agonists like LPS and PAM2CSK4 which, on the contrary, increased expression of RELA gene of the NF-kB pathway (Supplementary Fig. 2). It has been reported that expression of NFATc is inducible by calcium ionophore [48, 49], thus it is possible that calcium mobilization via SYK can trigger a positive loop for the activation of NFAT (Fig. 2F). This feature needs further studies but other authors have already suggested a relation between TREM2 and NFAT that, after translocation in the nucleus, co-operates with other transcription factors and proteins to regulate expression of immune related-genes [11].

NFAT and NFAT partners are activated via distinct signaling pathways and these interactions influence the locus of the binding to DNA and the outcome of the transcriptional regulation, thus providing a tuning mechanism of the biological functions. The NFAT genes appeared recently in the evolution in order to cope with the increasing complexity of the immune response in vertebrates, including control of inflammatory process triggered by PRRs and collaboration between innate and adaptive immunity [50]. For this reason, we tested the functional mechanism triggered by SULF A in the context of the inflammatory response of DC to LPS (Fig. 5A). Combination of the two stimuli produced a co-operative enhancement of the migration activity of h-MoDC in comparison to SULF A or LPS alone (Fig. 5B). Use of a specific inhibitor of NFAT significantly reduced the migration, thus corroborating the suggestion that this transcription factor is involved in the intracellular signaling of TREM2 leading to DC maturation. According to these results, SULF A did not reduce the LPS-dependent release of pro-inflammatory cytokines by *h*-MoDC but boosted synthesis of IL-10 (Fig. 5C) with a slight downregulation of the surface expression of CD86, CD83 and HLA-DR (Supplementary Fig. 10). This DC phenotype is different from that generated by SULF A alone and, in our opinion, is committed to the attenuation of the inflammatory stimulus. In fact, IL-10 is a regulatory cytokine that is produced with delayed kinetics compared to proinflammatory cytokines in order to prevent chronic inflammation and tissue damage due to an excessive immune response [37, 38]. It is worth noting that initiation of the anti-inflammatory pathway by LPS is driven by NFAT1 and NFAT3 in DCs [51], while production of IL-10 requires the contribution of NFATc members after dectin-1 engagement in macrophages and DCs [52]. This regulatory function of SULF A fits well with a general characteristic of TREM2 whose signalling is critically dependent on ligands, intracellular state and tissue context [11]. The mediated effect of SULF A on T cells in an allogeneic MLR further supports the differentiation of an unconventional DC phenotype mostly committed to the maintenance of homeostasis and tuning of the immune response. T cell priming is a hallmark of DC function and the MLR is a consolidated in vitro model for studying the mechanisms of allo-responsiveness. Thus, it is significant that SULF A increased expansion of DC-dependent T cells and synthesis of IL-4 and IL-2 (Fig. 5E–F) without affecting the levels of pro-inflammatory cytokines such as IL-12 and IFNγ (Fig. 5G–H).

In conclusions, we described that SULF A stimulates the unconventional maturation of a DC subtype with homeostatic or regulatory functions. This activity is mediated by the engagement of TREM2 and, according to the preliminary results, is downstream controlled by NFAT which can integrate other signaling pathways by co-operation with NFAT partners, e.g. NF-κB, in order to produce the regulatory outcome. In consideration of the role of DC in the positive or negative modulation of leukocyte response, the activity of SULF A may lay the groundwork for the development of a new class of drugs with therapeutic potential in chronic-inflammation and cancer [53–56]. Furthermore, SULF A is the first synthetic small molecule targeting TREM2 selectively. This finding warrants the investigation of this product in diseases well known to be bound to TREM-2 dysfunction, such as chronic and neurodegenerative diseases [11–14].

### Supplementary Information

Below is the link to the electronic supplementary material.

Supplementary file1 (DOCX 3152 KB)

## Data Availability

All data generated or analysed during this study are included in this published article and its supplementary information files. Further information is available from the corresponding author on reasonable request.
