# Supplementary material for: Sulfavant A as the first synthetic TREM2 ligand discloses a homeostatic response of dendritic cells after receptor engagement
Source: Cell Mol Life Sci. 2022 Jun 20;79(7):369. doi: 10.1007/s00018-022-04297-z (PMC9207826; doi:10.1007/s00018-022-04297-z)

**Supplementary Information**

**Identification of Sulfavant A as the first synthetic TREM2 ligand discloses a homeostatic response of Dendritic Cells after receptor engagement**

Carmela Gallo, Emiliano Manzo, Giusi Barra, Laura Fioretto, Marcello Ziaco, Genoveffa Nuzzo, Giuliana d'Ippolito, Francesca Ferrera, Paola Contini, Daniela Castiglia, Claudia Angelini, Raffaele De Palma and Angelo Fontana

This file includes:

Supplementary Figure 1 (Page 2)

Supplementary Figure 2 (Page 3)

Supplementary Figure 3 (Page 4)

Supplementary Figure 4 (Page 5)

Supplementary Figure 5 (Page 6)

Supplementary Figure 6 (Page 7)

Supplementary Figure 7 (Page 8)

Supplementary Figure 8 (Pages 9)

Supplementary Figure 9 (Page 10)

Supplementary Figure 10 (Page 11)

Supplementary Figure 11 (Page 12)

**Supplementary Figure 1**. Dose response curve and EC_50_ of SULF A for the activation of the response in TREM2 -reporter Cells.


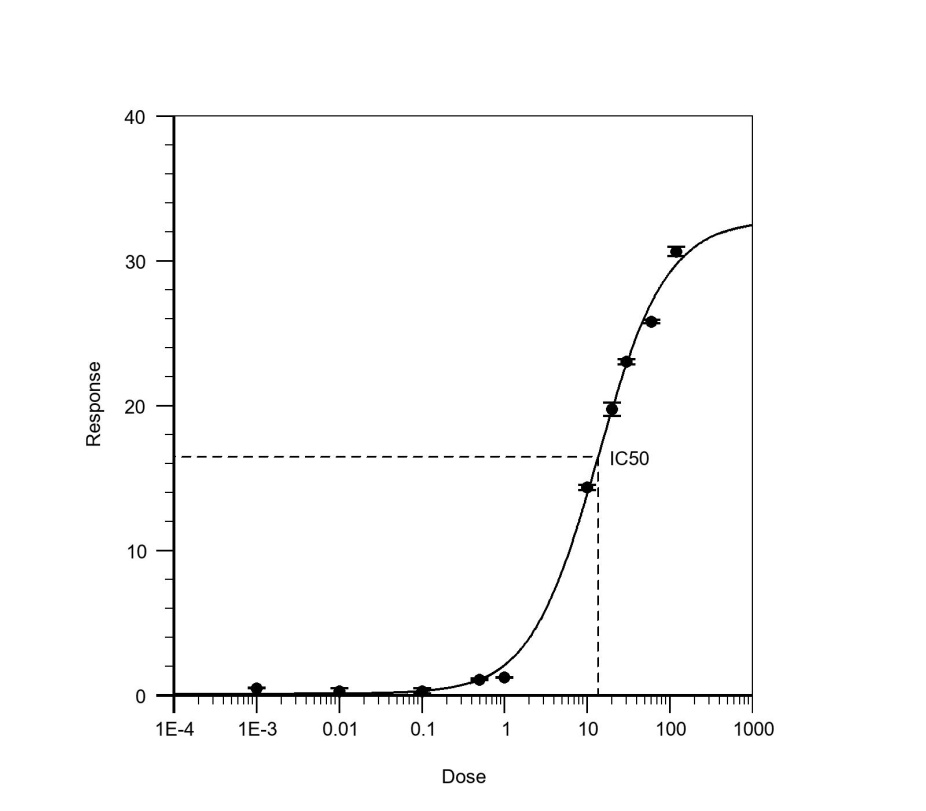


**Supplementary Figure 2**. Expression of the transcription factors RELA (**A**) and NF-κB p52 (**B**) in *h*-MoDC by treatment with the TLR2 and TLR4 agonists, PAM2CSK4 (25 ng/mL) and LPS (1 ng/mL). Gene levels analysis were performed at 6 different times from 45 minutes to 24 hours from the stimulation on cells obtained from n=4 matched donors. Ctrl= untreated cells. Statistical significance was assessed using a two-way RM ANOVA test, followed by a series of one-way RM ANOVA tests for each time point, with BH correction.


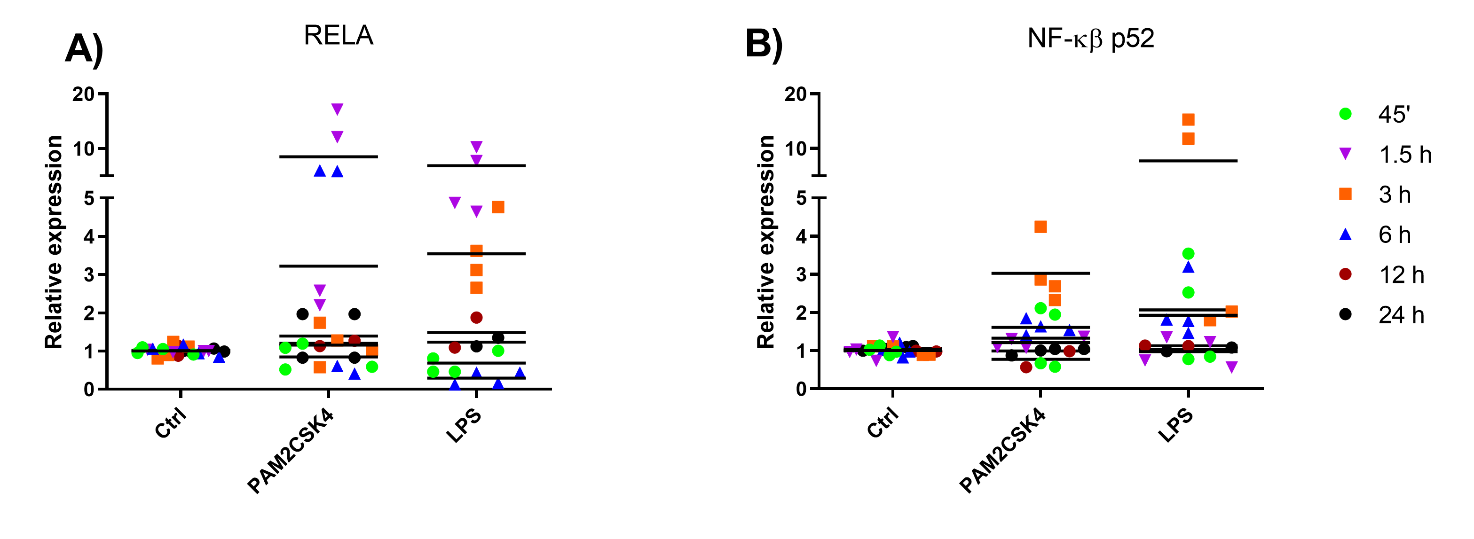


**Supplementary Figure 3**. Expression of DAP12 (*n=*2), SYK (*n=*2) and the transcription factors NF-κB p65 (RELA) (*n=*4), NF-κB p52 (*n=*4), NFAT-1(*n=*4) and NFAT-2 (*n=*4) in *h*-MoDC by treatment with 10 µg/mL SULF A. Gene levels analysis were performed at 6 different times from 45 minutes to 24 hours from the stimulation on cells obtained from n matched donors. Ctrl= untreated cells. Statistical significance was assessed using a two-way RM ANOVA test, followed by a series of one-way RM ANOVA tests for each time point, with BH correction.

**
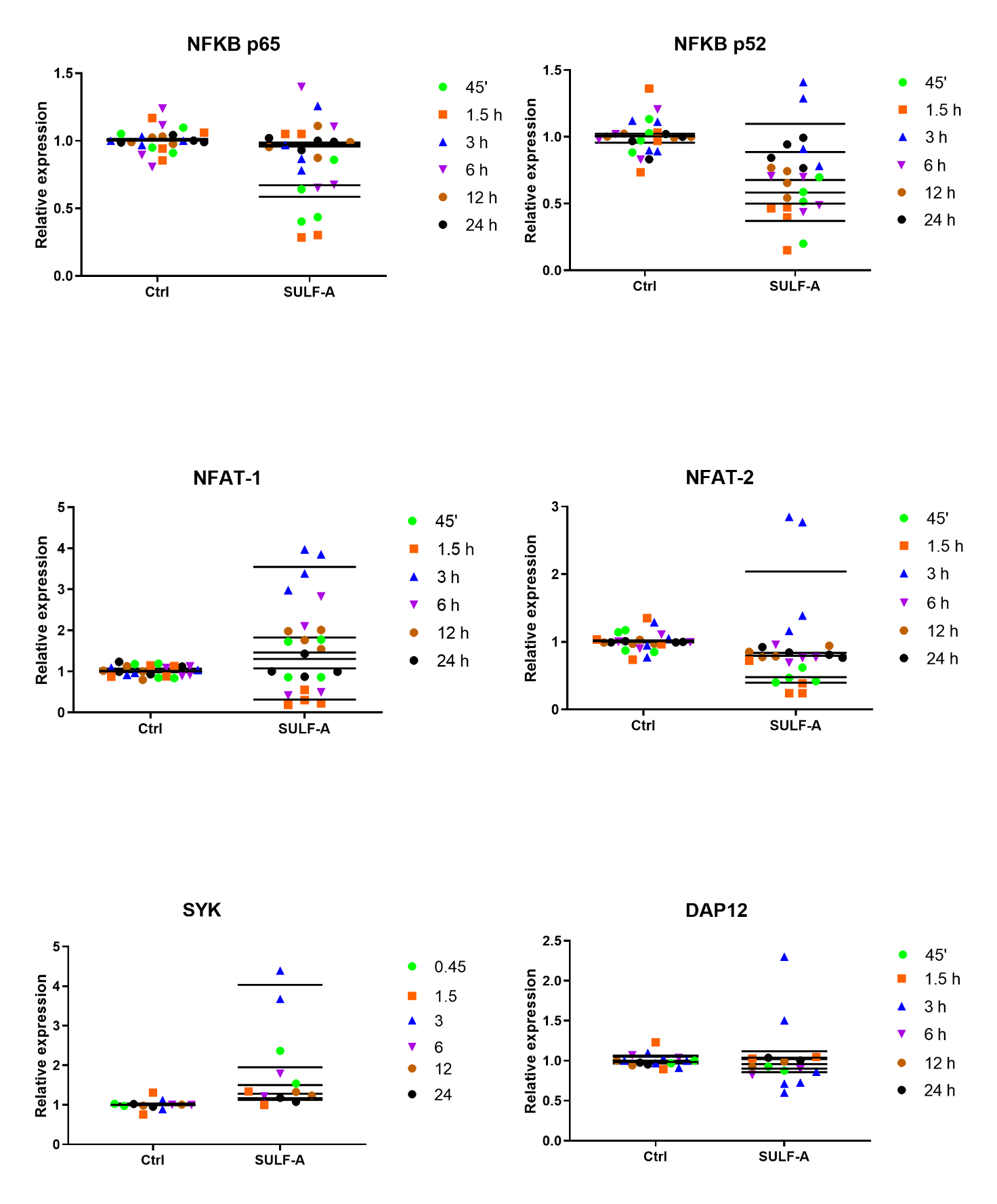
**

**Supplementary Figure 4.** Gating experiment displaying forward scatter (FSC) and HLA-DR, CD83 and CD86 expression of *h*-MoDCs by increasing concentration of anti-TREM2 blocking antibody (bAb). CTRL = untreated cells (upper left); 0.2 bAb = 0.2 µg/mL anti-TREM2 bAb; 0.5 bAb = 0.5 µg/mL anti-TREM2 bAb; 1 bAb = 1 µg/mL anti-TREM2 bAb.


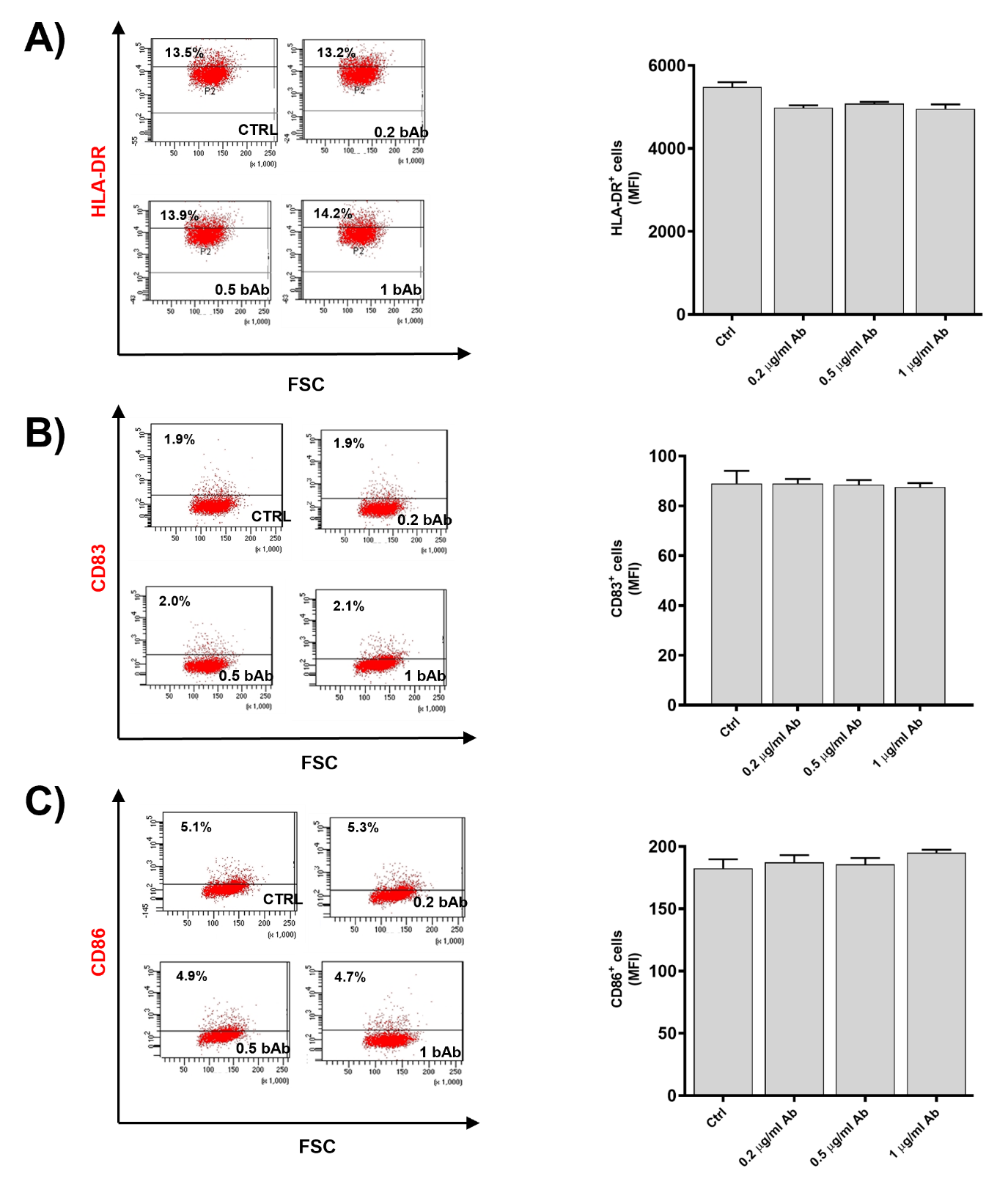


**Supplementary Figure 5**. Analysis by flowcytometry of the streptavidin on *h*-MoDCs at increasing concentration of the anti-TREM2 blocking antibody (bAb). Staining was performed by incubation with TREM2 anti-goat biotin secondary antibody (1:1000).. CTRL = untreated cells; 0.5 bAb = 0.5 µg/mL bAb; 1 bAb = 1 µg/mL bAb.


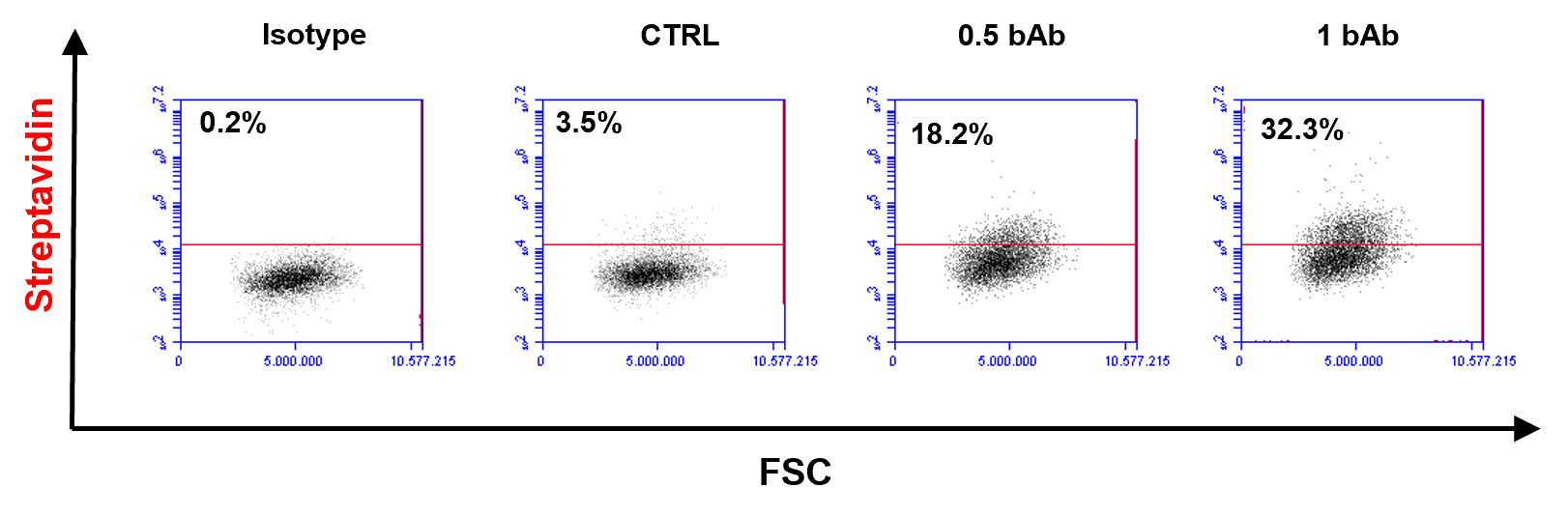


**Supplementary Figure 6**. Gating experiment displaying forward scatter (FSC) and CD86 (red spots) and CD83 (blue spots) expression on *h*-MoDCs treated by SULF A at increasing concentration of the anti-TREM2 blocking antibody (bAb). CTRL = untreated cells (upper left); SULF A = 10 µg/mL SULF A (upper right); 0.2 bAb+SULF A = 0.2 µg/mL bAb followed by 10 µg/mL SULF A (bottom left); 1 bAb+SULF A = 1 µg/mL anti-TREM2 bAB followed by 10 µg/mL SULF A (bottom right). The percentage on each plot is relative to CD83/CD86 double positive cell population.


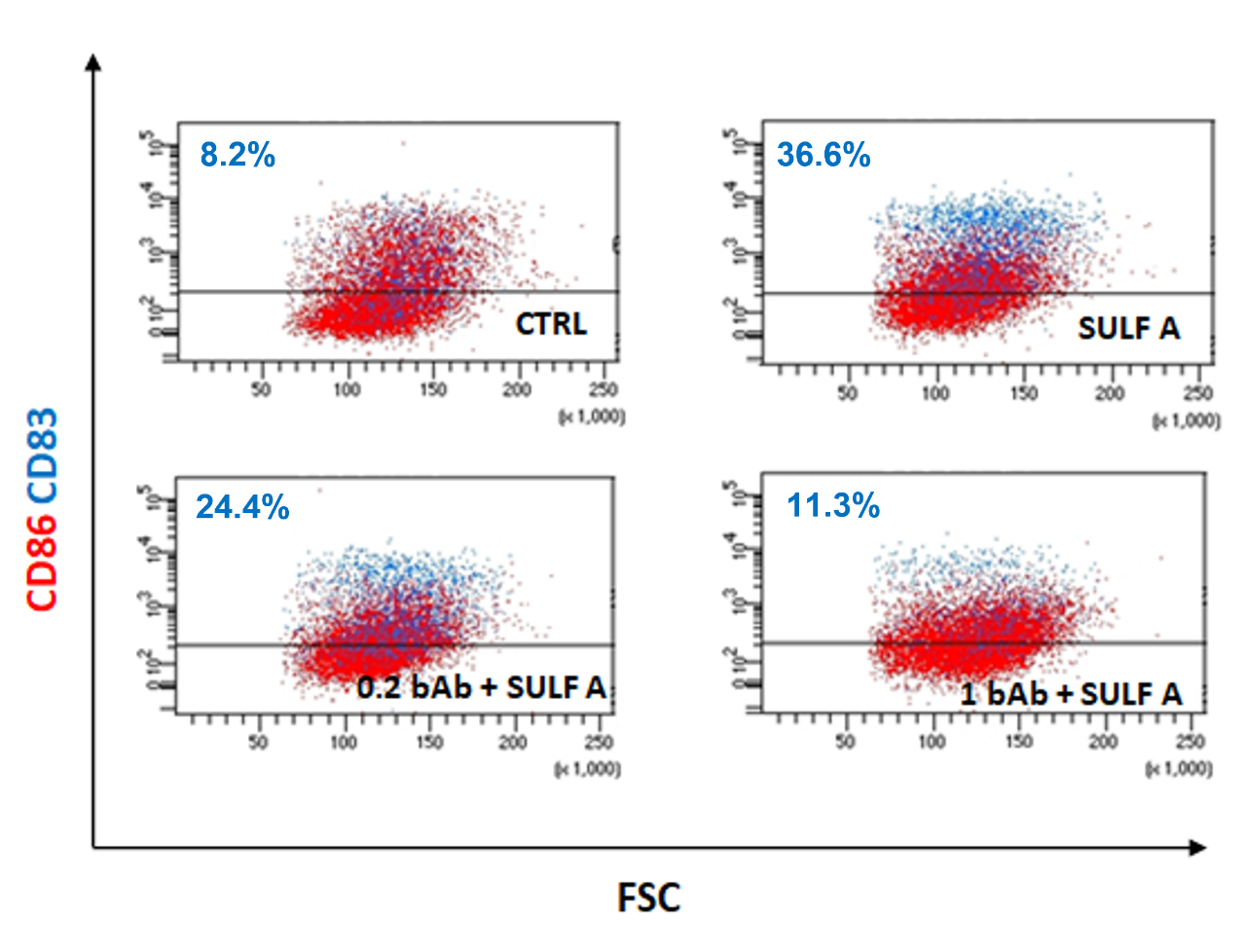


**Supplementary Figure 7**. Expression of surface maturation markers on *h*-MoDCs of n=3 matched donors by 10 µg/mL SULF A alone and together with increased concentrations of an isotypic control antibody (Ab; polyclonal normal goat IgG; AB-108-C; R&D Systems, Milan, Italy). (**A**) HLA DR (A); (**B**) CD83 (right); CD86 (left). Ctrl = untreated cells; SULF A = 10 µg/mL SULF A. One-way ANOVA with repeated measures followed by post hoc test. For HLA-DR ad CD83, two technical replicates were averaged before proceeding to the analysis. Statistical significance was assessed using one-way RM ANOVA followed by post-hoc analysis where all condition levels were compared to Ctrl using paired samples T-tests. BH correction was used in the post-hoc analysis.


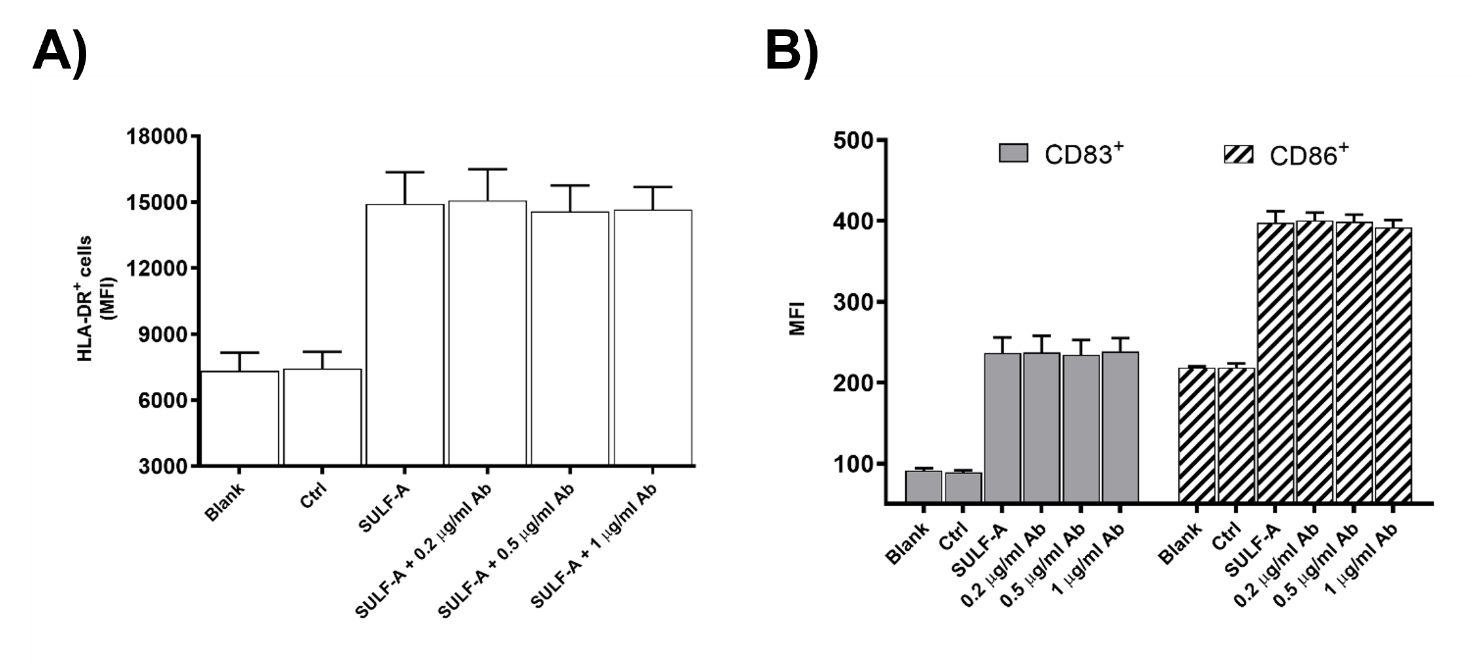


**Supplementary Figure 8**. Expression of the surface maturation markers HLA-DR, CD83 and CD86 on DCs by 200 nM negative siRNA and 200 nM TREM2 siRNA. Measurements were carried by flocytometry at 24 and 48 h. Isotypic control = untreated cells.


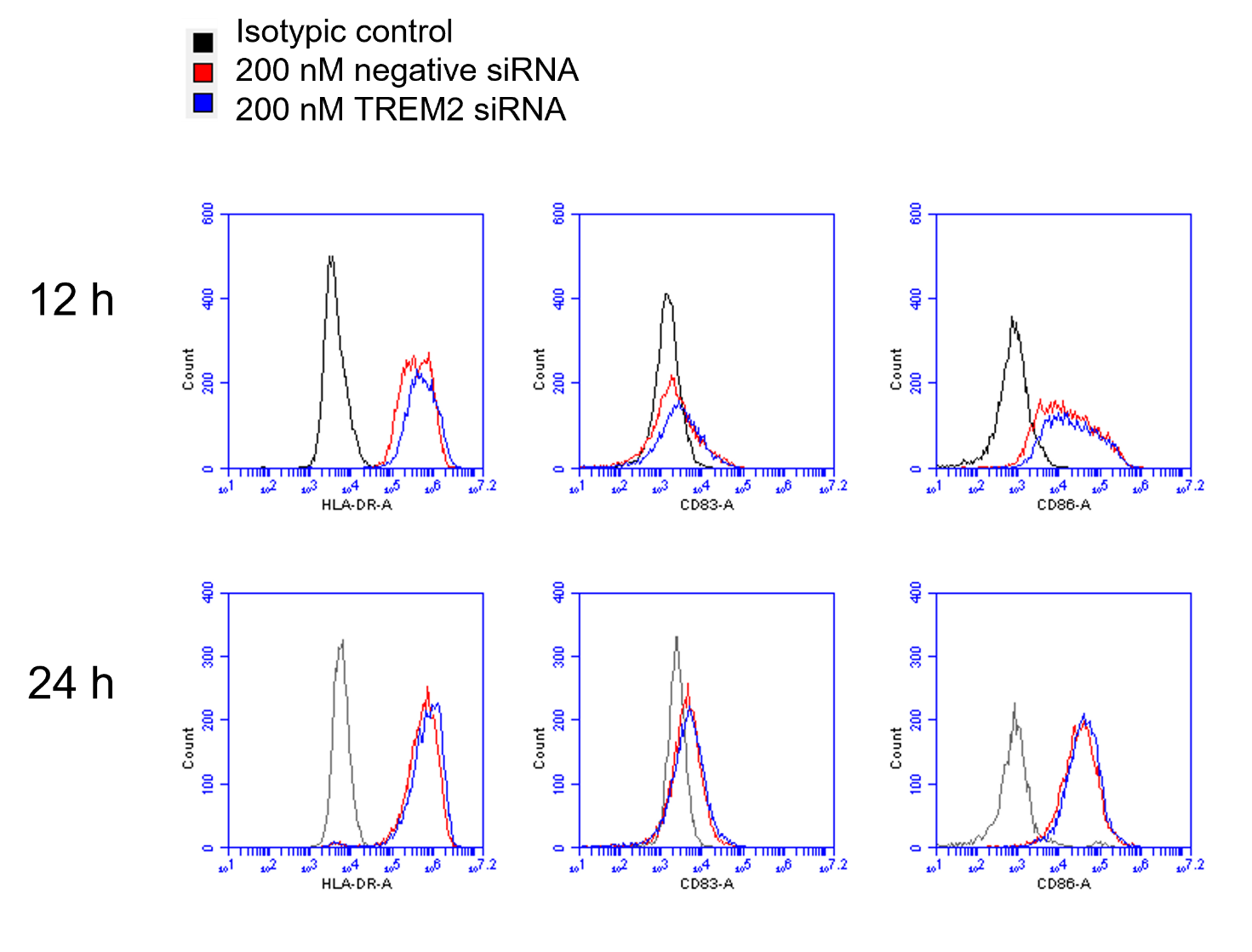


**Supplementary Figure 9**. Surface analysis by flowcytometry of TREM2 expression on *h*-MoDCs from n=3 matched donors (with 2 technical replicates) after 12 h from transfection with 200 nM TREM2 siRNA. cells were stained by TREM2- APC antibody. Data are expressed as percentage of TREM2 positive cells. Statistical analysis was carried out on the average of the two technical replicates by paired samples T-test with a two-side alternative. Significance: ** P < 0.01. CTRL = untreated cells.


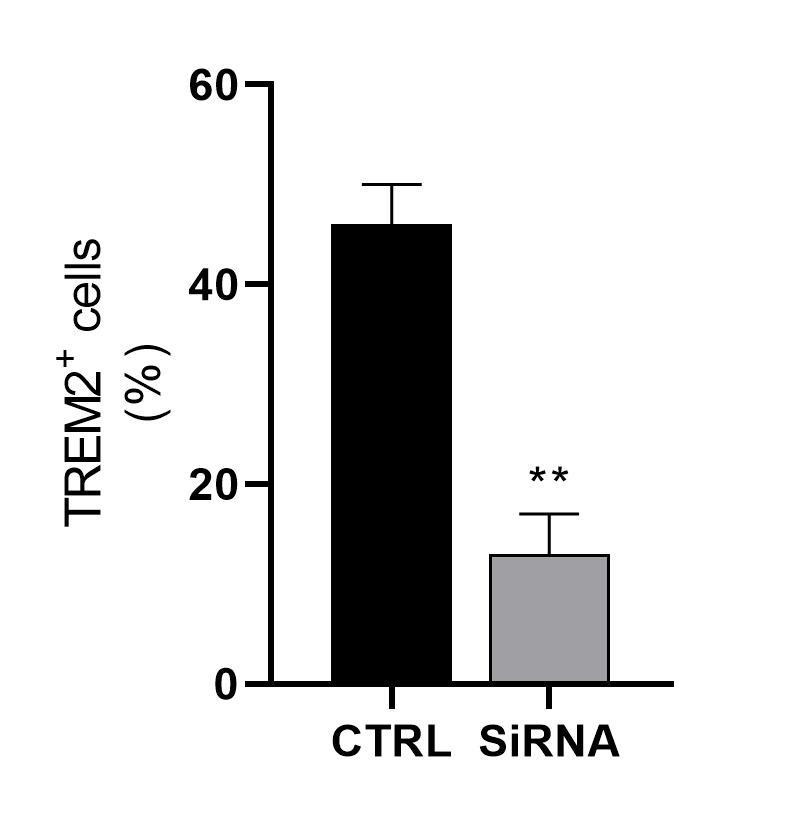


**Supplementary Figure 10**. Flowcytometry analysis of the expression of HLA-DR (**A**), CD83 (**B**) and CD86 (**C**) on the cell surface of *h*-MoDCs in co-stimulation experiments with 5 ng/mL LPS and 10 µg/mL SULF A. LPS = single treatment with 5 ng/mL; SULF A + LPS = treatment with 10 µg/mL SULF A followed after 30 min by 5 ng/mL LPS. The histograms on the right of each row tabulates the effects of LPS (red) and co-stimulation (blue) of SULF A plus LPS.


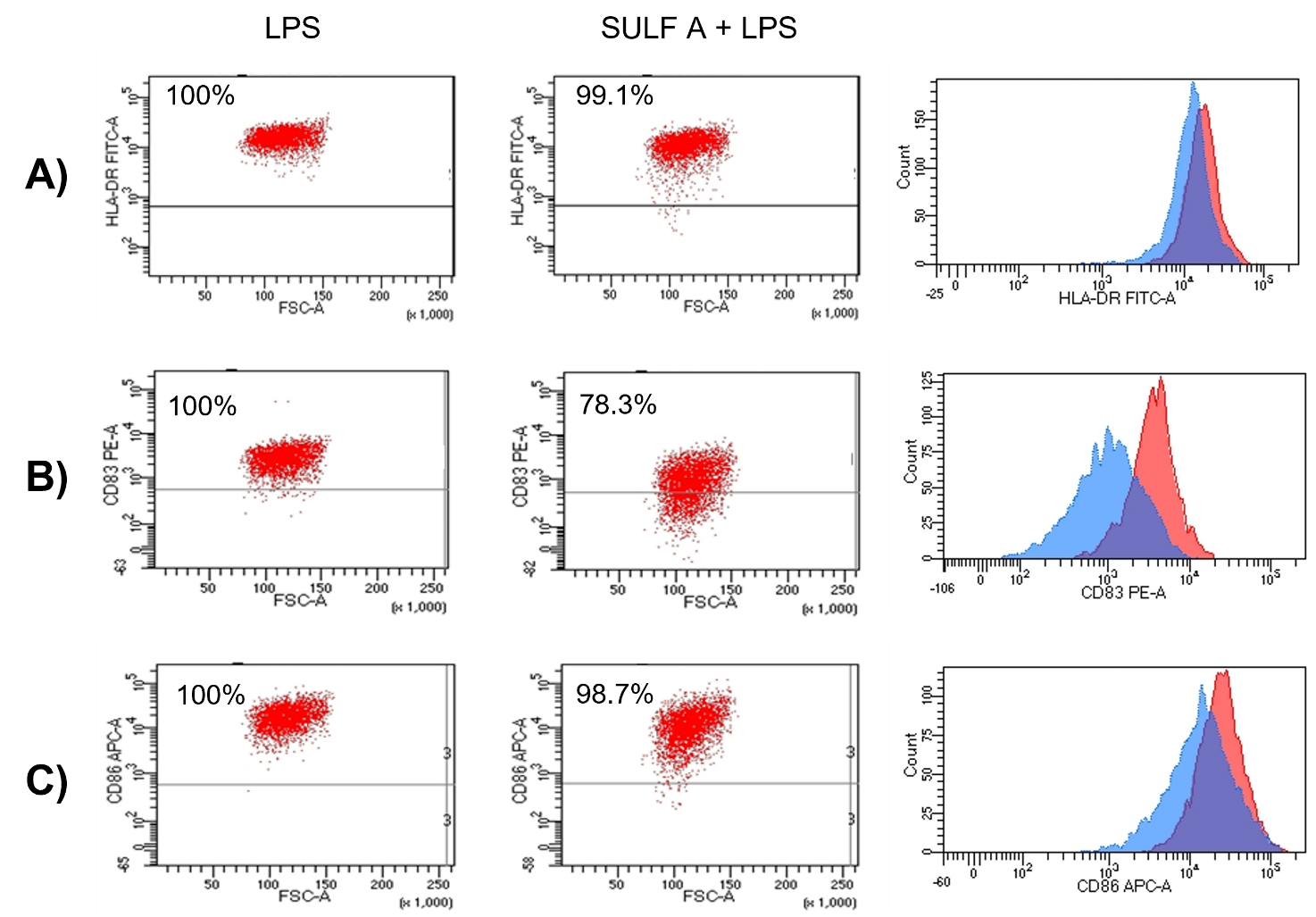


**Supplementary Figure 11**. Production of IL-2 and IL-4 measured by ELISA in the supernatants of Naïve T cells (1x 10^5^) cultured in the presence of anti CD3/CD28 beads for 7 days. The cytokine quantification was carried after 7 days from the stimulation by 10 µg/mL SULF A (n =4 matched donors). CTRL = untreated cells; SULF A = cells treated with 10 µg/mL SULF A. Statistical analysis was performed by the non-parametric paired samples Wilcoxon test (with two-side alternative). ns= data not statistically significant (p-value ˃ 0.05).


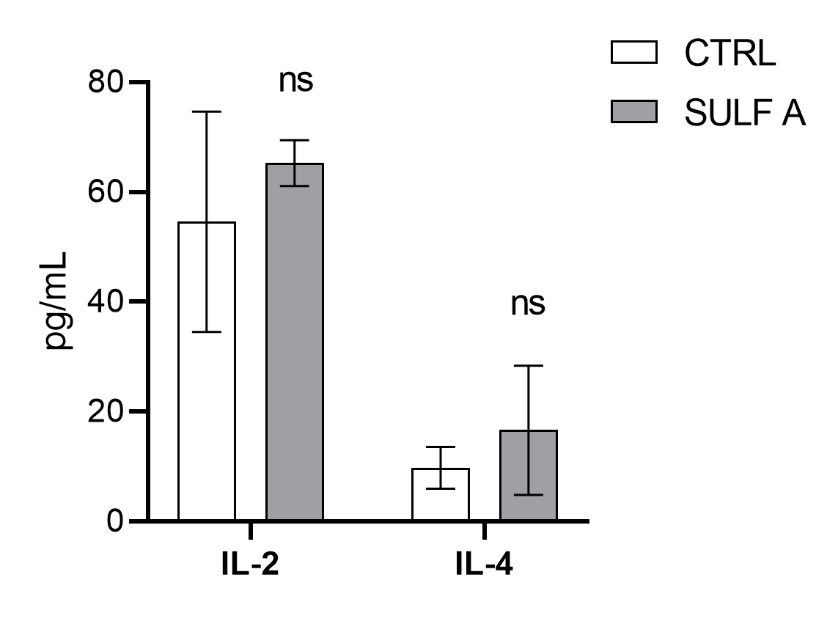

Supplement: Supplementary file 1 — Supplementary file1 (DOCX 3152 KB) [file 18_2022_4297_MOESM1_ESM.docx]
